# Surgeon-Performed Point-of-Care Ultrasound in the Diagnosis of Acute Sigmoid Diverticulitis: A Pragmatic Prospective Multicenter Cohort Study

**DOI:** 10.7759/cureus.33292

**Published:** 2023-01-03

**Authors:** Bogdan D Dumbrava, Hajar S Abdulla, Jorge Pereira, Alan Biloslavo, Mauro Zago, Jamal H Hashem, Nitya Kumar, Martin Corbally, Gary A Bass, Thomas N Walsh

**Affiliations:** 1 Department of Surgery, Connolly Hospital, Dublin, IRL; 2 Department of Surgery, Royal College of Surgeons in Ireland - Medical University of Bahrain, Busaiteen, BHR; 3 Department of Surgery, Tondela-Viseu Hospital Center, Viseu, PRT; 4 Department of Surgery, Cattinara University Hospital, Trieste, ITA; 5 Department of Surgery, Policlinico San Pietro, Ponte San Pietro, ITA; 6 Department of Epidemiology and Public Health, Royal College of Surgeons in Ireland - Medical University of Bahrain, Busaiteen, BHR; 7 Division of Traumatology, Surgical Critical Care and Emergency Surgery, University of Pennsylvania, Philadelphia, USA

**Keywords:** acute abdomen, ultrasound, abdominal sonography, diverticulitis, pocus

## Abstract

Background and purpose

Early diagnosis and risk stratification of sigmoid diverticulitis rely heavily on timely imaging. Computerized tomography (CT), the gold standard diagnostic test, may be delayed due to resource constraints or patient comorbidity. Point-of-care ultrasound (POCUS) has an established role in trauma evaluation, and could potentially diagnose and stage acute diverticulitis, thus shortening the time to definitive treatment.

Aims

This study aimed to benchmark the accuracy of surgeon-performed POCUS against CT in diagnosing and staging acute diverticulitis. A secondary aim was to evaluate the duration between the POCUS and the confirmatory CT scan report.

Patients and methods

A pragmatic prospective multicenter cohort study (ClinicalTrials.gov Identifier: NCT02682368) was conducted. Surgeons performed point-of-care ultrasound as first-line imaging for suspected acute diverticulitis. POCUS diagnosis and radiologic Hinchey classification were compared to CT as the reference standard.

Results

Of 45 patients with suspected acute diverticulitis, POCUS classified 37 (82.2%) as uncomplicated diverticulitis, four (8.8%) as complicated diverticulitis, and four (8.8%) as other diagnoses. The POCUS-estimated modified radiologic Hinchey classification was largely concordant with CT staging with an accuracy of 88.8% (95% CI, 75.95-96.2%), a sensitivity of 100% (95% CI, 90.2- 100%) and a specificity of 44.4% (95% CI, 13.7-78.8%). The positive predictive value (PPV) was 87.8% and the negative predictive value (NPV) was 100%. There was moderate agreement between CT and POCUS, with a Cohen’s kappa coefficient of 0.56. The mean delay between CT and POCUS was 9.14 hours (range 0.33 to 43.5).

Conclusion

We examined the role of POCUS in the management of acute diverticulitis and our findings suggest that it is a promising imaging modality with the potential to reduce radiation exposure and treatment delays. Adding a POCUS training module to the surgical curriculum could enhance diagnosis and expedite the management of acute diverticulitis.

## Introduction

Early accurate diagnosis and stage-based risk stratification of acute sigmoid diverticulitis rely heavily on timely diagnostic imaging [1-3]. Computerized tomography (CT) is the gold standard, but it may be delayed due to resource constraints or patient comorbidity, especially during out-of-hours presentations, in COVID pandemic-affected institutions and in resource-limited regions. Delays in diagnosis will delay the commencement of appropriate treatment and may negatively impact morbidity, length of stay, and overall healthcare cost [4]. Portable ultrasound machines are now almost ubiquitous in emergency departments. Clinicians increasingly use ultrasound for focused purposes such as peripheral arterial or venous access, central venous catheter insertion, focused assessment with sonography for trauma (FAST) scanning for intra-abdominal hemorrhage, and obstetric fetal assessment [5-7]. To date, uptake by surgeons in diagnosing other abdominal emergencies has been limited [8]. Specifically, its role in acute diverticulitis, where prompt diagnosis and management are essential, has not been embraced by general surgeons [9].

An aging global population with the increasing prevalence of obesity, smoking, low fiber diet, and better access to radiographic diagnostics, have contributed to a rising incidence of hospital presentations for acute sigmoid diverticulitis [10,11]. Hospitals worldwide are facing rising costs and greater demand for resources. These challenges, especially in resource-constrained health systems, necessitate a new algorithm for diagnosing and managing acute diverticulitis that is safe, efficient, and cost-effective [10,12]. Over-reliance on CT may introduce delays in care, particularly for patients with comorbidities or in resource-poor environments where availability may be time-bound, limited, or even absent. CT also exposes the patient to ionizing radiation risk [13]. Over the last decade, several studies have reported that, with technological developments, the accuracy, sensitivity, and specificity of ultrasound are improving leading to its increased adoption as a first-line imaging modality [14-18]. In most of these studies, however, the context differed as a stand-alone diagnostic examination was performed by radiologists or sonographers rather than coupled with the history and physical examination at the point of care. Outside of the primary survey of the trauma patient, there is little experience of POCUS use by surgeons in the emergency setting. 

The aim of this study was to assess the accuracy of surgeon-performed ultrasound compared to CT scanning in diagnosing and staging acute diverticulitis in the adult population. A secondary aim was to evaluate the duration between the POCUS and the confirmatory CT scan report.

## Materials and methods

Study design & patient recruitment

This study was conducted in four centers in Italy, Portugal, and Ireland. Forty-five patients that fulfilled the eligibility criteria were recruited. Patients presenting to the emergency department with clinical suspicion of acute diverticulitis were recruited for the study. Following clinical assessment, a POCUS-trained surgeon performed the ultrasound examination. All four surgeons (BD, JP, AB, MZ) had three or more years of experience in point-of-care sonography and were certified tutors for the Modular Ultrasound ESTES® Course [19]. 

Following the POCUS assessment, patients were either to be admitted for CT assessment or for surgery or were to be discharged if the ultrasound examination was negative in the context of limited and isolated left iliac fossa pain, no other symptoms, and no laboratory abnormalities. Further radiological investigations were requested as per local policies. Standardization of pre-imaging preparation, or contrast usage, was not required. 

The radiologists conducting the CT examinations were blinded to the POCUS results. Both the POCUS and CT reports were uploaded to the National Integrated Imaging System (NIMIS) for storage and retrieval of medical imaging [20].

Inclusion criteria

All patients aged 18 years or older and suspected of having acute diverticulitis were considered eligible. Clinical suspicion of acute diverticulitis was based on an appropriate history, clinical findings, and laboratory support. A clinical history was considered indicative of diverticulitis if the patient presented with left lower abdominal pain, with or without constipation, diarrhea, or rectal bleeding, coupled with the physical findings of fever and left iliac fossa tenderness. A raised C-reactive protein (CRP) and leucocytosis added further support for the inclusion of acute diverticulitis in our differential. Patients with left iliac fossa pain and even one of the clinical findings such as left iliac fossa tenderness, rectal bleeding, fever (oral, rectal, or axillary temperatures in excess of 38°C), change in bowel habit or elevation in one of the laboratory parameters (either CRP or WBC) were included in the study.

Exclusion criteria

Patients with a known history of diverticulitis, patients with a previous colorectal resection, or patients with an increased body habitus (BMI ≥30) were excluded from the study. Of patients presenting without the said exclusion criteria, we did not include patients with non-specific abdominal pain without a change in bowel habit, patients without left iliac fossa tenderness, and patients who were apyrexial or had a normal C-reactive protein or normal neutrophil counts.

POCUS procedure and definitions

A sonographic evaluation was performed using commercially available portable scanners with a low-frequency convex probe (2.5-6 Mhz) and a high-frequency linear probe (up to 13 or 15 Mhz). Panoramic views were performed with the convex probe searching for free fluid, collections, etc. and the linear probe, with better resolution, was used to focus specifically on the bowel wall. As all patients were admitted emergently and examined in the emergency department, no special bowel preparation was considered.

Diverticulitis was diagnosed on POCUS based on any one of the following criteria: bowel wall thickness ≥ 4mm, pain on graded compression, pericolic fat changes (increased echogenicity), and diverticula; while abscess, fistula, the presence of free fluid and the absence of peristalsis defined complicated diverticulitis [21]. 

A modified Hinchey classification [22] based on POCUS findings was used: 0 - mild diverticulitis, Ia - localized pericolic inflammation/phlegmon, Ib - localized pericolic abscess, II - pelvic or distant abdominal abscess, III - purulent peritonitis, and IV - fecal peritonitis. The primary diagnosis was categorized as uncomplicated diverticulitis, complicated diverticulitis, or other diagnoses.

Data collection

Patient data were securely stored behind a firewalled server in full compliance with the EU General Data Protection Regulation 2016/679, utilizing the REDCap® (Research Electronic Data Capture) platform [23], which is a secure, web-based application supporting data capture for research studies. The REDCap server and webpage for this study were hosted on the MUSEC® online e-learning platform.

Parameters such as age, gender, record ID, surgeon’s initials, and country were recorded on the datasheet. The POCUS findings were labeled with a hospital identification number, distinctive for each patient. The patients’ names, surnames, home addresses, or dates of birth were not uploaded to the REDCap servers to ensure patient confidentiality and anonymity. Each surgeon involved in the study had a username and password and access was restricted to only the data uploaded by that specific center.

Ethical approval

The study protocol was registered with ClinicalTrials.gov under the ID NCT02682368. The Connolly Hospital Ethics Committee authorized (approval number NCT02682368) this study based on national healthcare criteria. All patients were fully informed, and written consent was acquired. Each study site obtained local institutional ethics review board approval for the study protocol.

Statistical analysis

Data extracted from REDCap® database Version 8.2.2 were statistically analyzed using JamoviStats Version 0.9.5.2. Sensitivity, specificity, predictive values, likelihood ratios and accuracy for POCUS in establishing Hinchey classification and confirming the diagnosis compared to reference values were calculated. The reference value was the formal findings in the CT radiological report. The 95% confidence intervals were computed using the efficient-score method [24,25]. The positive and negative predictive values of POCUS were also calculated. Cohen’s Kappa coefficient was calculated to measure inter-observer agreement between POCUS and the formal radiology report. Finally, an estimation of the study’s post-hoc power was conducted.

## Results

Demographics

Forty-five patients [31 females (68.9%)] presenting with lower abdominal pain and a clinical diagnosis of acute diverticulitis underwent a POCUS examination. The mean age was 61.3 years (range 28 to 93). Forty-four (98%) patients required admission and further radiological investigations and one patient was discharged with an outpatient follow-up. None of the patients required surgical intervention.

Clinical manifestations

Tenderness in the left iliac fossa was the most common finding at presentation, occurring in 37 patients (82%), increased CRP in 33 (73%), white cell count (WCC) elevation in 21(47%), fever in 19 (42%) localized peritonism in 11 (24%) and rectal mucosal discharge or bleeding in six (13%).

Diagnosis, staging, and POCUS findings

Following clinical assessment and laboratory investigations, a POCUS examination was performed, and the findings are listed in Table 1.

**Table 1:** Frequency of acute diverticulitis features on POCUS POCUS: Point-of-care ultrasound

POCUS findings	N=45 (%)
Pericolic changes (increased echogenicity)	39 (86.7%)
Pain on graded compression	36 (80.0%)
Bowel wall thickness >4mm	28 (62.2%)
Diverticulae	25 (55.5%)
Absence of Peristalsis	3 (6.7%)
Presence of free fluid	2 (4.4%)
Hinchey Stage	
Hinchey 0	11 (24.4%)
Hinchey Ia	26 (57.7%)
Hinchey Ib	4 (8.88%)
Hinchey II	0 (0%)
Hinchey III	0 (0%)
Hinchey IV	0 (0%)

Pericolic fat changes, pain on graded compression, and increased bowel wall thickness were the most common POCUS features identified in this patient cohort. Diverticula were identified in 25 (55.6%) patients, whereas complications (e.g abscess, free fluid) were only seen in two (4%) patients.

The clinical impression was of uncomplicated diverticulitis in 37 patients (82.2%), complicated diverticulitis in four (8.8%), and four patients (8.8%), clinically considered to have acute diverticulitis, were classified as “other diagnoses” following POCUS evaluation. In three of these, the POCUS diagnosis was of gynecological pathology, which was confirmed by formal ultrasonography. One patient with a 9 cm section of colonic mural thickening, loss of haustra, and echogenic pericolic fat, without evidence of diverticula, had a diagnosis of colitis confirmed by CT.

Of the 41 patients with POCUS-diagnosed acute diverticulitis, their POCUS staging identified 11 as Hinchey 0, 26 as Hinchey Ia, and four as HincheyIb (Table 1). Evidence of Hinchey II, Hinchey III, or Hinchey IV was identified on none of the scans.

Accuracy of POCUS versus CT

Using CT as the reference standard, there were 36 true positive findings, four true negatives, and five false positives where CT detected a different pathology. There were no false-negative diagnoses. POCUS sensitivity was 100% (95% CI, 90.2-100%) with 44.4% specificity (95% CI, 13.7-78.8%). POCUS had an overall accuracy of 88.8% (95% CI, 75.95-96.2%). The positive predictive value (PPV) was 87.8% and the negative predictive value (NPV) was 100%. Cohen’s kappa coefficient was calculated as 0.56, which confirms a moderate agreement between radiology and POCUS regarding the overall diagnosis.

False positives and discordant cases

The five false positive cases where POCUS had diagnosed diverticulitis, were diagnosed on CT as colitis in 3, terminal ileitis in one, and one case of diverticulosis with pancreatic cancer. For Hinchey staging, POCUS and CT agreed in 27 of 36 (75%) instances. There were nine discordant cases (25%) where POCUS underestimated the stage in five patients and overestimated it in four. 

Interval between POCUS and CT report

Between POCUS and the formal radiology CT report, the mean duration (in hours) was calculated as 9.14 hours (median 4.5 hours) with a range of up to 43.5 hours. 

Post-hoc power estimation

Post-hoc power for our study sample of 45 participants was calculated and found to be 85%, based on a conservative estimate. This was done by assuming one false negative to enable the prerequisite calculation of a non-zero odds ratio.

## Discussion

This study found that point-of-care ultrasound had a sensitivity of 100% in identifying acute diverticulitis in the emergency setting. There was no significant difference in accuracy between POCUS and CT in diagnosing acute diverticulitis. The sensitivity of POCUS in this study confirmed the findings of a higher sensitivity compared to CT in some [26,27] but not all studies [28,29]. The specificity of POCUS, however, was low in our study at 44%. This could be explained by the small study size as other studies, with larger sample sizes, reported specificities of approximately 90% [14,28,29]. Specific features such as thickening of the bowel wall, pericolic fat changes, and pain on graded compression were the main findings on sonography described in other studies making these the key features [15,28,30-34]. Along with these essential elements, other findings such as diverticula, and the absence of peristalsis or abscesses aided in the classification of severity as reported by others [35,36].

The average interval between POCUS and the formal radiology CT report was 9.14 hours, ranging up to 43.5 hours. Arguably, many of those patients requiring urgent operative management are clinically apparent but not all, as highlighted by a recent high-profile case where a fatal outcome might have been avoided by the availability of POCUS [37]. Accepting that many patients “will need a CT anyway”, we suggest that the real utility of POCUS lies in triaging undifferentiated symptomatic patients into those with Hinchey I diverticulitis who need no further diagnostic studies and who, perhaps, could be discharged from the ED, and identifying Hinchey II+ patients who may benefit from admission for CT-guided drainage. Although 25% of our patients were misclassified on POCUS the clinical outcome was unaffected in these early stages of uncomplicated diverticulitis (1a and 1b) as fluid resuscitation, bowel rest, pain relief, antibiotics, and anti-inflammatory medications were indicated regardless of which early Hinchey stage [38]. A recent study by Zago et al. found that POCUS correctly identified the Hinchey classification in 93% of acute diverticulitis cases [30]. 

The role of CT as a reference value for POCUS can be questioned as it too carries a risk of false positives and negatives [39]. To improve diagnostic accuracy, the absolute reference value would need to be the gross appearance at the surgery or on the histopathology report [31,40-42], but access to these reference values presents infrequently and takes time to accumulate. One study suggests that the lower sigmoid colon may be challenging to assess by transabdominal ultrasound as diverticulitis deep in the pelvis cannot be ruled out by ultrasound, especially if performed with an empty bladder [14]. Our study had no false-negatives patients, but this may be due to the study population size. The final diagnosis ultimately rests on the complete clinical picture comprising patient history, clinical manifestations, and special investigations. CT is essential if other diagnoses, such as pancreatic cancer (in one patient) are not to be missed. An argument can be made for CT screening for everyone over a certain age to detect occult disease. Having made the diagnosis, the choice of CT or POCUS for follow-up of patients that fail to improve on standard management needs to be considered. Caputo et al. [43] have suggested that POCUS should be repeated at 72 hours if there is no evidence of early and significant clinical improvement. The more conventional view is that sonography should be followed by a CT scan [29,44], but once the patient has recovered, POCUS may have a role in clinical follow-up [21], avoiding excessive radiation exposure. With increased experience and studies such as this, the respective roles of CT and ultrasound will be clarified in due course. 

Our study has a number of weaknesses. The authors are POCUS enthusiasts and regularly teach the Modular Ultrasound for Surgeons ESTES Course (MUSEC) [45,46], and the accuracy of POCUS performed by non-enthusiasts may not match these results, but that applies equally to clinical examination. There is a learning curve for POCUS, and educational performance benchmarks occur at variable points for image interpretation for different examination types which must be considered when developing training standards for POCUS credentialing [14,47]. Our conclusions are limited in generalizability due to a small number of surgeons trained to perform POCUS to the required standard in each study center and by our decision to exclude patients with a known diagnosis of diverticulitis or obesity. However, these ‘proof of concept’ pilot data are hypothesis-generating and future work may include prospective validation of surgeon POCUS proficiency in the diagnosis of acute diverticulitis (compared with CT as gold-standard) following a standardized training program (such as the MUSEC course) [19]. Future studies should involve more POCUS-certified surgeons in a larger cohort, but this will require POCUS training programs embedded in the surgical training curriculum [48]. A further issue is that the radiologists reporting on the CTs were all generalists while, ideally, specialist radiologists with similar expertise would have provided reports on the reference values. It could be argued that the actual patient diverticulitis population is not accurately reflected in this study as we excluded obese patients from this study because high-density fat is a limitation to sonographic detection of distinguishable features while obesity is an associated factor for acute diverticulitis [49]. The outcomes in our study reflect a real-world working environment since the scans were carried out by frontline surgeons, either in-training or experienced surgeons on duty, and the post-hoc power of 85% increases our confidence that the results of our study are scalable worldwide.

## Conclusions

In conclusion, POCUS proved to be fast and effective in diagnosing uncomplicated diverticulitis, allowing these patients to be managed conservatively. While CT has an undeniable role in diagnosing complex diseases and detecting extracolonic pathology, we believe that given its high sensitivity, POCUS should have a role as a triaging imaging modality in the emergency department to expedite treatment, reduce costs and avoid unnecessary radiation exposure. With increasing technical sophistication, allied to artificial intelligence, the role of POCUS may expand in the future to a first-line imaging modality, pending its evaluation in a sufficiently powered study.

## References

[1] Boermeester MA, Humes DJ, Velmahos GC, Søreide K (2016). Contemporary review of risk-stratified management in acute uncomplicated and complicated diverticulitis. World J Surg.

[2] van Dijk ST, Bos K, de Boer MG (2018). A systematic review and meta-analysis of outpatient treatment for acute diverticulitis. Int J Colorectal Dis.

[3] Lembcke B (2016). Ultrasonography in acute diverticulitis - credit where credit is due. Z Gastroenterol.

[4] Manabe N, Haruma K, Nakajima A, Yamada M, Maruyama Y, Gushimiyagi M, Yamamoto T (2015). Characteristics of colonic diverticulitis and factors associated with complications: a Japanese multicenter, retrospective, cross-sectional study. Dis Colon Rectum.

[5] Lee L, DeCara JM (2020). Point-of-care ultrasound. Curr Cardiol Rep.

[6] Barjaktarevic I, Kenny JS, Berlin D, Cannesson M (2021). The evolution of ultrasound in critical care: from procedural guidance to hemodynamic monitor. J Ultrasound Med.

[7] Algodi M, Wolfe DS, Taub CC (2022). The utility of maternal point of care ultrasound on labor and delivery wards. J Cardiovasc Dev Dis.

[8] Whitson MR, Mayo PH (2016). Ultrasonography in the emergency department. Crit Care.

[9] Nielsen K, Richir MC, Stolk TT, van der Ploeg T, Moormann GR, Wiarda BM, Schreurs WH (2014). The limited role of ultrasound in the diagnostic process of colonic diverticulitis. World J Surg.

[10] O'Grady M, Turner G, Currie W, Yi M, Frizelle F, Purcell R (2020). Acute diverticulitis: an ongoing economic burden on the health system. ANZ J Surg.

[11] Hupfeld L, Pommergaard HC, Burcharth J, Rosenberg J (2018). Emergency admissions for complicated colonic diverticulitis are increasing: a nationwide register-based cohort study. Int J Colorectal Dis.

[12] Sullivan JF, do Brasil (Res) M, Roman JW, Milder EA, Carter E, Lennon RP (2021). Utility of point of care ultrasound in humanitarian assistance missions. Mil Med.

[13] Shokoohi H, Nasser S, Pyle M, Earls JP, Liteplo A, Boniface K (2020). Utility of point-of-care ultrasound in patients with suspected diverticulitis in the emergency department. J Clin Ultrasound.

[14] Dirks K, Calabrese E, Dietrich CF (2019). EFSUMB position paper: recommendations for gastrointestinal ultrasound (GIUS) in acute appendicitis and diverticulitis. Ultraschall Med.

[15] Lembcke BJ, Strobel D, Dirks K, Becker D, Menzel J (2015). Statement of the section internal medicine of the DEGUM - ultrasound obtains pole position for clinical imaging in acute diverticulitis. Ultraschall Med.

[16] Cuomo R, Barbara G, Pace F (2014). Italian consensus conference for colonic diverticulosis and diverticular disease. United European Gastroenterol J.

[17] Helou N, Abdalkader M, Abu-Rustum RS (2013). Sonography: first-line modality in the diagnosis of acute colonic diverticulitis?. J Ultrasound Med.

[18] Sartelli M, Catena F, Ansaloni L (2016). WSES Guidelines for the management of acute left sided colonic diverticulitis in the emergency setting. World J Emerg Surg.

[19] Zago M, Martinez Casas I, Pereira J (2016). Tailored ultrasound learning for acute care surgeons: a review of the MUSEC (Modular UltraSound ESTES Course) project. Eur J Trauma Emerg Surg.

[20] Smith J, Kok HK, Torreggiani WC (2016). Examining the end-user experience of the National Integrated Medical Imaging System (NIMIS). Irish Medical Journal.

[21] Nazerian P, Gigli C, Donnarumma E (2021). Diagnostic accuracy of point-of-care ultrasound integrated into clinical examination for acute diverticulitis: a prospective multicenter study. Ultraschall Med.

[22] Barat M, Dohan A, Pautrat K (2016). Acute colonic diverticulitis: an update on clinical classification and management with MDCT correlation. Abdom Radiol (NY).

[23] Harris PA, Taylor R, Thielke R, Payne J, Gonzalez N, Conde JG (2009). Research electronic data capture (REDCap)--a metadata-driven methodology and workflow process for providing translational research informatics support. J Biomed Inform.

[24] Sullivan KM, Dean A, Soe MM (2009). OpenEpi: a web-based epidemiologic and statistical calculator for public health. Public Health Rep.

[25] Altman D, Machin D, Bryant T, Gardner M Statistics with Confidence: Confidence Intervals and Statistical Guidelines, 2nd Edition. Gardner. Statistics with confidence.

[26] Liljegren G, Chabok A, Wickbom M, Smedh K, Nilsson K (2007). Acute colonic diverticulitis: a systematic review of diagnostic accuracy. Colorectal Dis.

[27] Kandagatla PG, Stefanou AJ (2018). Current status of the radiologic assessment of diverticular disease. Clin Colon Rectal Surg.

[28] Laméris W, van Randen A, Bipat S, Bossuyt PM, Boermeester MA, Stoker J (2008). Graded compression ultrasonography and computed tomography in acute colonic diverticulitis: meta-analysis of test accuracy. Eur Radiol.

[29] Andeweg CS, Wegdam JA, Groenewoud J, van der Wilt GJ, van Goor H, Bleichrodt RP (2014). Toward an evidence-based step-up approach in diagnosing diverticulitis. Scand J Gastroenterol.

[30] Zago M, Biloslavo A, Mariani D, Pestalozza MA, Poillucci G, Bellio G (2021). Surgeon-performed ultrasound for the staging of acute diverticulitis: preliminary results of a prospective study. J Trauma Acute Care Surg.

[31] Schwerk WB, Schwarz S, Rothmund M (1992). Sonography in acute colonic diverticulitis. A prospective study. Dis Colon Rectum.

[32] King WC, Shuaib W, Vijayasarathi A, Fajardo CG, Cabrera WE, Costa JL (2015). Benefits of sonography in diagnosing suspected uncomplicated acute diverticulitis. J Ultrasound Med.

[33] Parulekar SG (1985). Sonography of colonic diverticulitis. J Ultrasound Med.

[34] Pradel JA, Adell JF, Taourel P, Djafari M, Monnin-Delhom E, Bruel JM (1997). Acute colonic diverticulitis: prospective comparative evaluation with US and CT. Radiology.

[35] Blaivas M, Kirkpatrick AW, Rodriguez-Galvez M, Ball CG (2009). Sonographic depiction of intraperitoneal free air. J Trauma.

[36] Coppolino F, Gatta G, Di Grezia G (2013). Gastrointestinal perforation: ultrasonographic diagnosis. Crit Ultrasound J.

[37] Dyer C (2013). Surgeon jailed for manslaughter after postponing surgery on man with perforated bowel. BMJ.

[38] Andeweg CS, Mulder IM, Felt-Bersma RJ (2013). Guidelines of diagnostics and treatment of acute left-sided colonic diverticulitis. Dig Surg.

[39] van Randen A, Laméris W, van Es HW (2011). A comparison of the accuracy of ultrasound and computed tomography in common diagnoses causing acute abdominal pain. Eur Radiol.

[40] Ritz JP, Lehmann KS, Loddenkemper C, Frericks B, Buhr HJ, Holmer C (2010). Preoperative CT staging in sigmoid diverticulitis--does it correlate with intraoperative and histological findings?. Langenbecks Arch Surg.

[41] Wilson SR, Toi A (1990). The value of sonography in the diagnosis of acute diverticulitis of the colon. AJR Am J Roentgenol.

[42] Fozard JB, Armitage NC, Schofield JB, Jones OM (2011). ACPGBI position statement on elective resection for diverticulitis. Colorectal Dis.

[43] Caputo P, Rovagnati M, Carzaniga PL (2015). Is it possible to limit the use of CT scanning in acute diverticular disease without compromising outcomes? A preliminary experience. Ann Ital Chir.

[44] Ripollés T, Sebastián-Tomás JC, Martínez-Pérez MJ, Manrique A, Gómez-Abril SA, Torres-Sanchez T (2021). Ultrasound can differentiate complicated and noncomplicated acute colonic diverticulitis: a prospective comparative study with computed tomography. Abdom Radiol (NY).

[45] Marconi M, Mariani D, La Greca A (2019). Not only FAST The MUSEC® experience in training surgeons. Ann Ital Chir.

[46] Pereira J, Bass GA, Mariani D (2020). Surgeon-performed point-of-care ultrasound for acute cholecystitis: indications and limitations: a European Society for Trauma and Emergency Surgery (ESTES) consensus statement. Eur J Trauma Emerg Surg.

[47] Blehar DJ, Barton B, Gaspari RJ (2015). Learning curves in emergency ultrasound education. Acad Emerg Med.

[48] Khan MA, Abu-Zidan FM (2020). Point-of-care ultrasound for the acute abdomen in the primary health care. Turk J Emerg Med.

[49] Makar M, Pisano TJ, Xia W, Greenberg P, Patel AV (2021). The impact of obesity on mortality and clinical outcomes in patients with acute diverticulitis in the United States. J Gastrointestin Liver Dis.

